# The Impact of Cognitive Behavioral Therapy on Peripheral Interleukin-6 Levels in Depression: A Systematic Review and Meta-Analysis

**DOI:** 10.3389/fpsyt.2022.844176

**Published:** 2022-05-13

**Authors:** Haijing Ma, Jiatong Xu, Ruonan Li, Roger S. McIntyre, Kayla M. Teopiz, Bing Cao, Fahui Yang

**Affiliations:** ^1^Key Laboratory of Cognition and Personality, Faculty of Psychology, Ministry of Education, Southwest University, Chongqing, China; ^2^Mood Disorders Psychopharmacology Unit, Toronto, ON, Canada; ^3^Department of Psychiatry, University of Toronto, Toronto, ON, Canada; ^4^Department of Pharmacology, University of Toronto, Toronto, ON, Canada; ^5^National Demonstration Center for Experimental Psychology Education, Southwest University, Chongqing, China

**Keywords:** cognitive behavioral therapy, IL-6, depression, inflammation, cytokines, biomarkers

## Abstract

There is interest in the role of peripheral interleukin-6 (IL-6) in depression and the effect of treatment (e. g., pharmacologic, psychosocial, neurostimulation). However, the relationship between cognitive behavioral therapy (CBT), IL-6 and depression has not yet been established. We conducted a meta-analysis to explore the association between CBT and change of peripheral IL-6 levels in depressive symptoms or major depressive disorder (MDD). A systematic search of online databases (i.e., PubMed, Web of Science, Google Scholar, PsycINFO, and Cochrane Library) was completed from inception to May 2021. In total, 10 eligible papers with 940 participants reporting peripheral IL-6 levels before and after CBT were included in the analysis. The main result indicates that peripheral levels of IL-6 were significantly lower after CBT intervention in individuals with depression, with a small effect (SMD = 0.38, 95% CI: 0.07, 0.69, *p* = 0.02). The results of subgroup analyses demonstrate that (1) there was a significant decrease in IL-6 for studies that were equal to or <8 weeks in duration vs. more than 8 weeks in duration, and (2) IL-6 was significantly reduced in the Diagnostic and Statistical Manual of Mental Disorders (DSM) diagnosis (i.e., DSM-IV, DSM-IV-TR, or DSM-V) of MDD, but not for the subgroup without DSM diagnosis. Publication year was identified as a potential contributor to heterogeneity of the results from our analysis. Taken together, our findings support the notion that CBT influences peripheral IL-6 in individuals with depression and represents a point of commonality with other antidepressant treatment modalities (e.g., antidepressants).

**Systematic Review Registration:**

https://doi.org/10.17605/osf.io/tr9yh, identifier: 10.17605/osf.io/tr9yh.

## Introduction

Major depression is a major public health issue implicating significant economic and psychosocial burden (1). Current predictions reported by the World Health Organization (WHO) indicate that depression will be the leading cause of disease burden globally by 2030 (1). The pathophysiology of depression is unknown, but is known to be factorial involving neurobiological systems subserving stress response, and their interaction with environmental, psychosocial, and spatial determinants of health (2). A study by (3) reported that major depression is associated with activation of the inflammatory response system (4). It is also reported that an activated inflammatory system is associated with abnormal hedonic and cognitive function in adults with depression (5–7).

It is separately reported that disparate antidepressant modalities influence peripheral cytokine concentrations in adults with depression (6). Moreover, it is separately reported that elevated inflammatory markers may be a predictor of non-response with selective serotonin reuptake inhibitor (SSRI) therapy (2, 8). However, previous studies have reported mixed results as it relates to the relationship between depression and inflammatory biomarkers (9–11). Due to the heterogeneity of inflammatory markers as a group, it has been suggested to explore the effect of a single inflammatory marker, such as IL-6, in depression (12–14). Excess or chronic inflammatory cytokine activity, on the other hand, disrupts various neural activities, including neurotransmitter signaling, neurotransmitter synthesis, reuptake, and release (15–17). Thus, neurocircuit function, especially that linked to mood and cognition, is affected (18).

Cognitive behavioral therapy (CBT) is a structured, short-term and targeted psychotherapy with replicated evidence of acute antidepressant and recurrence prevention effects in adults with MDD. Cognitive behavioral therapy is one of the most common psychosocial interventions for mental disorders (19, 20). Cognitive behavioral therapy targets cognitions (i.e., thoughts) that reinforce dysfunctional beliefs and behaviors relevant to clinical symptoms (20, 21).

The mechanism of action of CBT is unknown and its effect on neurobiological systems implicated in depression are not well-characterized. The latest meta-analysis paper revealed that psychosocial interventions including CBT were significantly associated with levels of proinflammatory cytokines or markers (22). However, the study only included two papers on the topic of association between CBT and immune system function in people with depression. Besides, depression was not precisely defined in the study. Preliminary evidence suggests that peripheral IL-6 levels in adults with depression are reduced after 7 weeks of CBT in responder analysis (23). Results are mixed, insofar as a 16-week study of CBT monotherapy in adults with a first-episode of depression was associated with reduced TLR-4 signaling, but the changes from baseline to endpoint in TLR-2 signaling, IL-6, and c-reactive protein (CRP) levels were not statistically significant. A systematic review by Lopresti et al. (24) evaluating the association between CBT and change of peripheral IL-6 levels was not able to identify a consistent effect. For more rigorous verification, a meta-analysis evaluating the impact of CBT on changes of peripheral IL-6 levels in individuals with depression is urgent needed.

Against a background of inconsistent findings in the extant literature, the aim of the study herein was to use a meta-analysis to comprehensively and systematically evaluate the impact of CBT on changes of peripheral IL-6 levels in individuals with depression. The results of this analysis are intended to guide further mechanistic research and inform conceptual frameworks.

## Methods

### Search Strategy

Two investigators (HJM and JTX) independently conducted the literature search to identify studies reporting IL-6 levels of subjects with depression before and after CBT intervention. The information in this review was compiled by searching online databases: PubMed, Web of Science, Google Scholar, PsycINFO, Cochrane Library databases, and by searching the reference lists of relevant papers to locate additional studies that were not identified by the database searches. The databases were scanned from inception to May 2021. Systematic searches were completed using terms including “cognitive behavioral therapy,” “CBT,” “psychotherapy,” “inflammation,” “IL-6,” “interleukin,” and “immunity,” “MDD,” “major depressive disorder,” “depressive.”

### Eligibility Criteria

The inclusion criteria were as follows: (1) The study subjects were adults (≥18 years old) with a diagnosis of major depressive disorder (MDD) based on Diagnostic and Statistical Manual of Mental Disorders (DSM) criteria (i.e., DSM-IV, DSM-IV-TR, or DSM-V), or MDD with other chronic diseases; Or had a verified scale to assess depressive symptoms (including subthreshold depression: clinically relevant depressive symptoms, without meeting criteria for a full-blown MDD); (2) Follow-up results after CBT intervention were reported (baseline assessment scores vs. post-treatment scores); (3) The peripheral IL-6 levels were evaluated before and after CBT; (4) identified random controlled trials (RCT), open-label studies, and longitudinal studies with pre-test-post-test design were included in our analysis.

Exclusion criteria included: (1) Non-original studies (e.g., review, meta-analysis, systematic review, standards, guidelines, teaching materials, books; (2) Non-research articles (e.g., descriptive introduction of disease progression, etiology, intervention, differential diagnosis, research protocol); (3) Conference abstracts and unpublished literature; (4) case reports, case studies, case series studies, case control studies; (5) Basic experimental research studies (e.g., animal, cell, tissue, etc.); (6) studies including subjects that did not have a DSM-defined diagnosis of MDD, and/or included case groups with comorbid mental disorder diagnoses (e.g., schizophrenia, bipolar disorder) without depressive symptoms; (7) studies without follow-up results (8) studies including healthy individuals or individuals with other diseases analyzed as the control group; (9) studies that did not report on peripheral IL-6 levels as part of the study outcome.

### Outcome and Recorded Variables

The purpose for the meta-analysis was to examine the relationship between CBT and the change of peripheral IL-6 levels in individuals with depression from baseline to endpoint. First author, published year, country, sex (male/female), mean age (age range), total *N* at baseline, clinical diagnosis, comorbidity, IL-6 measure method, diagnostic criteria, study design and duration of intervention were recorded for each eligible study for analysis. All eligible studies were screened and evaluated by two independent investigators. We also carefully verified data from each included article to ensure the accuracy of the extracted data. Any discrepancies were resolved by discussion among all of the authors.

### Statistical Analysis

Statistical analysis was performed using Review Manager 5.4.1 and Stata 12.0. Forest plots were used to estimate the change of peripheral IL-6 levels in individuals with depression from baseline to end point, which was evaluated by the standardized mean difference (SMD) within a 95% confidence interval (CI). According to the statistical power analysis for the behavioral sciences (2nd edition), the effect size of SMD is judged using the following rules: trivial (SMD < 0.20), small (0.20 ≤ SMD < 0.50), medium (0.50 ≤ SMD <0.80), and a large effect (SMD ≥ 0.80). The chi-square and I-Squared (*I*^2^) test was used to evaluate the heterogeneity across the studies. It has been suggested that the adjectives low, moderate, and high (heterogeneity) be assigned to *I*^2^ values of 25, 50, and 75%. If *P* <0.10 or *I*^2^>50%, there would be a high degree of heterogeneity with statistical differences (25), and a random effects model was applied to pool data. The fixed effects meta-analysis was used in the other cases. To identify probable causes of heterogeneity, subgroup analyses about the development levels of countries, publication years, whether DSM diagnosed and duration of CBT intervention were carried out.

Meta-regression analysis was also performed to examine whether IL-6 levels in subjects with depression could be influenced by pre-specified independent variables, which evaluated the effect of years of publication, mean ages, and sex ratios. Sensitivity analysis was performed to identify potential outliers by eliminating each study individually, which examined the impact of each study on the overall effect size. Publication bias was assessed by applying Egger's test and Begg's test for funnel plot asymmetry.

We used Grades of Recommendations Assessment, Development and Evaluation (GRADE) to assess the quality of included studies (26). The assessments were based on following aspects: study limitations, risk of bias, inconsistency of results, indirectness (i.e., different subjects, interventions and results from the aimed ones), random error and publication bias. The total grade scored ≥0 indicated high-grade evidence, that scored −1 indicated moderate-grade evidence, that scored −2 indicated low-grade evidence, that scored ≤ -3 indicated very low-grade evidence. A two-tailed *P* <0.05 were considered significant in all test.

## Results

### Search Results

**Figure 1:**
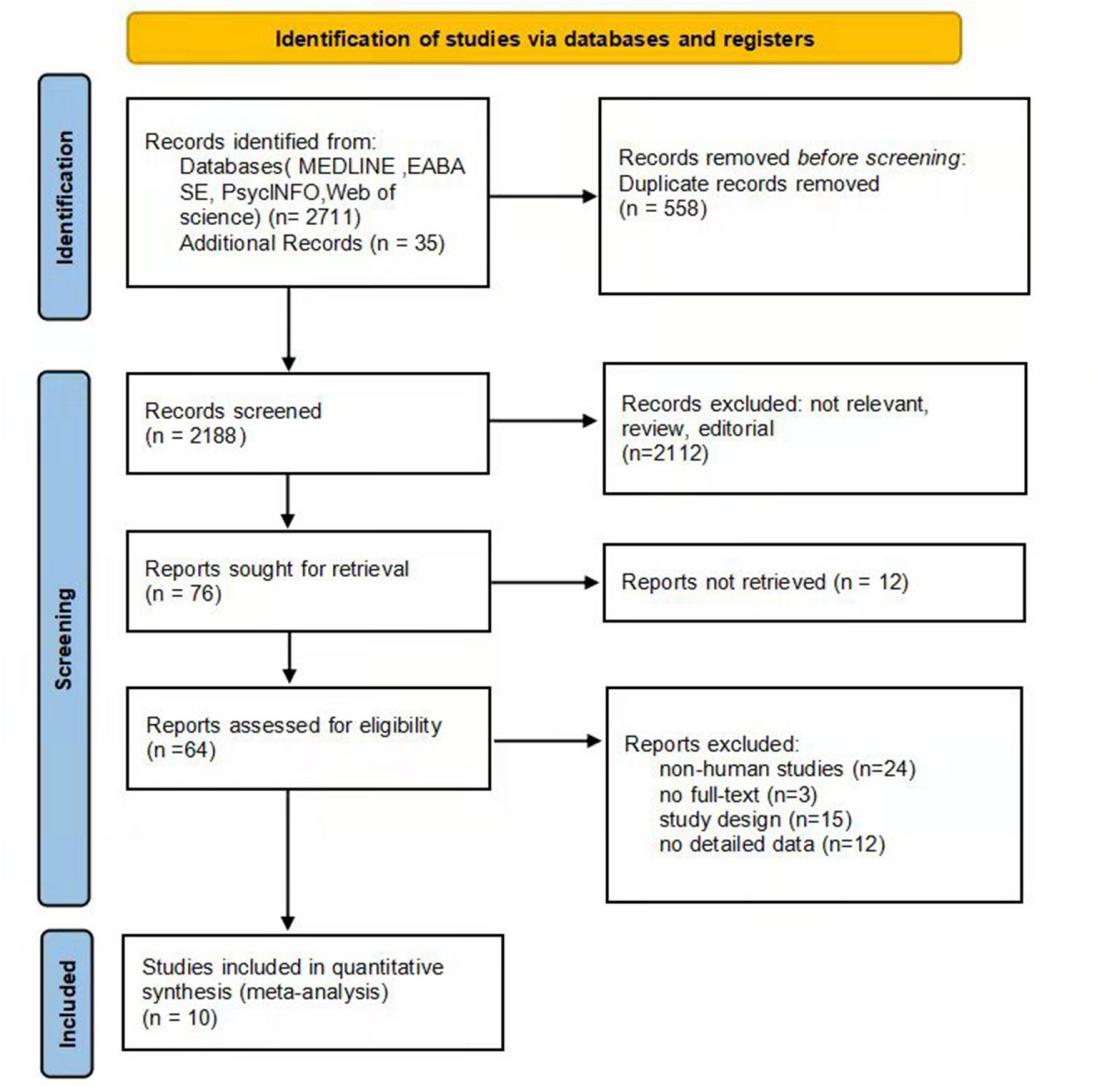
Summary of the article extraction process, including the reasons for exclusion.

The summary of the article extraction process for this meta-analysis is shown in Figure 1. In total, 2,746 records were identified as potentially eligible through the initial systematic literature search. After removing duplicate studies, 2,188 studies remained, of which 2,112 were further excluded based on reviews of titles and abstracts. Afterwards we examined the full text of the remaining 76 relevant articles, and 12 of them were not retrieved. In the remaining 64 papers, 24 were non-human studies, 3 were not full-text, 15 did not meet a criterion for inclusion, and 12 did not provide detailed data. Finally, 10 studies were eligible for inclusion in our meta-analysis (23, 27–35).

Ten studies were identified comprising 940 participants were conducted in six countries: USA (*n* = 3); Germany (*n* = 2); Brazil (*n* = 2); and China (*n* = 1), Sweden (*n* = 1), Hungary (*n* = 1). Six studies used Diagnostic and Statistical Manual of Mental Disorders IV (DSM-IV) as diagnostic criteria for depression; two implemented the Center for Epidemiological Survey (CES-D) and one study each used the Positive and Negative Affect Schedule, Hospital Anxiety, and Depression Scale (HADS), respectively. Eight studies had more females than males, among them, one had only female; one study had less females than males; and one did not include the sex. According to the GRADE system, four studies are rated moderate and six are rated HIGH. The main characteristics of the included studies and quality assessments are shown in Table 1.

**Table 1:** Characteristics of included studies.

**References**	**Country**	**Sex (Male/Female)**	**Mean age (Age range)**	**Total *N* at baseline**	**Clinical diagnosis**	**Comorbidity**	**IL-6 measure method**	**Diagnostic criteria**	**Study design**	**Duration**	**Quality assessments**
Moreira et al. (23)	Brazil	7/21	24.46 ± 3.61	97	MDD	No	Commercial mmunoassay kit	DSM-IV	Double-blind, Randomized trial	7 w*	Moderate
Euteneuer et al. (28)	German	18/16	36.9	101	MDD	Anxiety disorders Somatoform disorders	Flow cytometry using bead-based assays	DSM-IV	Double-blind, Randomized trial	16 w	High
Gazal et al. (29)	Brazil	0/11	25.18 ± 3.51	11	MDD	No	IL-6 immunoassay kit	DSM-IV	before-after study in the same patient	7w	Moderate
Kéri et al. (32)	Hungary	19/31	22.6	80	MDD	No	High-sensitivity enzyme-linked immunosorbent assay kits	DSM-IV and SCID-CV and HAM-D	Double-blind, Randomized trial	16 w	High
Zautra et al. (35)	USA	46/97	52.41	144	Depressed	Rheumatoid arthritis	Commercially available enzyme linked immunosorbent assay kits	DSM-IV	Randomized trial	8 w	High
Berk et al. (27)	USA	23/44	52.5	132	MDD	One or more chronic medical illnesses	Millipore's multiplexed high sensitivity cytokine magnetic bead-based immunoassay kits	DSM–IV and BDI-II	Double-blind, multi-site randomized clinical trial	12 w	High
Hsu et al. (31)	China		75.3 (±4.61)	20	Depressed	No	Not mentioned	CES-D	Randomized trial	8 w	Moderate
Hermanns et al. (30)	German	46/60	43.2 ± 14.9	214	Depressed	Diabetes	Quantikine HS (IL-6) ELISA kits	CES-D	Blind, Randomized Study		High
Moore et al. (34)	USA	9/40	70.86	100	Depressed	Dementia	ELISA	Positive and Negative Affect Schedule	Double-blind, Randomized trial	6 w	High
Lasselin et al. (33)	Sweden	9/32	40.9	41	Depressed	Longstanding pain	ELISA	HADS	Randomized trial	12 w	Moderate

* *w, weeks.*

*MDD, major depressive disorder; ELISA, high-sensitive enzyme-linked immunosorbent assays; DSM-IV, diagnostic and statistical manual of mental disorders IV; CES-D, center for epidemiological survey; HADS, Hospital anxiety and depression scale; BDI-II, beck depression inventory II; HAM-D, Hamilton depression scale; SCID-CV, structured clinical interview for DSM-IV axis I disorders -clinical version*.

### Preliminary Meta-Analysis Analysis Results

**Figure 2:**
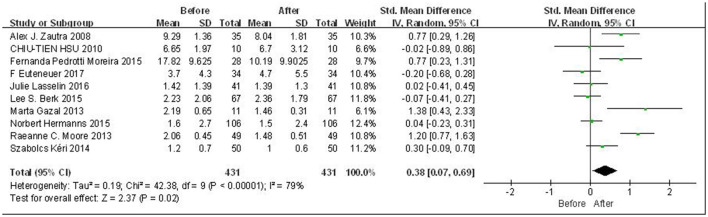
Forest plot for change in IL-6 before and after CBT.

Overall meta-analysis results are shown in Figure 2. Due to the high heterogeneity among included literatures (*p* < 0.00001; *I*^2^ = 79%), the random effects model was adopted for the analysis. Since the scales are consistent across studies, we used SMD for data processing. The results indicate that there was a statistically significant difference in the peripheral levels of IL-6 before and after CBT intervention, with a small effect (SMD = 0.38, 95% CI: 0.07, 0.69, *p* = 0.02). In order to explore the potential sources of heterogeneity, subgroup analysis, regression analysis, and sensitivity analysis were carried out in the following studies.

### Subgroup Analysis

We performed the subgroup analyses to identify the sources of the literature heterogeneity. For the classification of developed and developing countries, we found that the overall combined change of peripheral IL-6 levels was statistically significant in developing countries (SMD = 0.70, 95% CI: 0.02, 1.38, *p* = 0.04), but the data heterogeneity remained medium (*p* = 0.10, *I*^2^ = 57%). In the subgroup of developed countries, IL-6 levels do not have a significant overall comprehensive effect (*p* = 0.11), and its heterogeneity is also large (*p* < 0.0001, *I*^2^ = 82%), indicating that subgroup analysis of the classification of developed and developing countries cannot explain the heterogeneity sources of IL-6 levels in all included studies.

**Figure 3:**
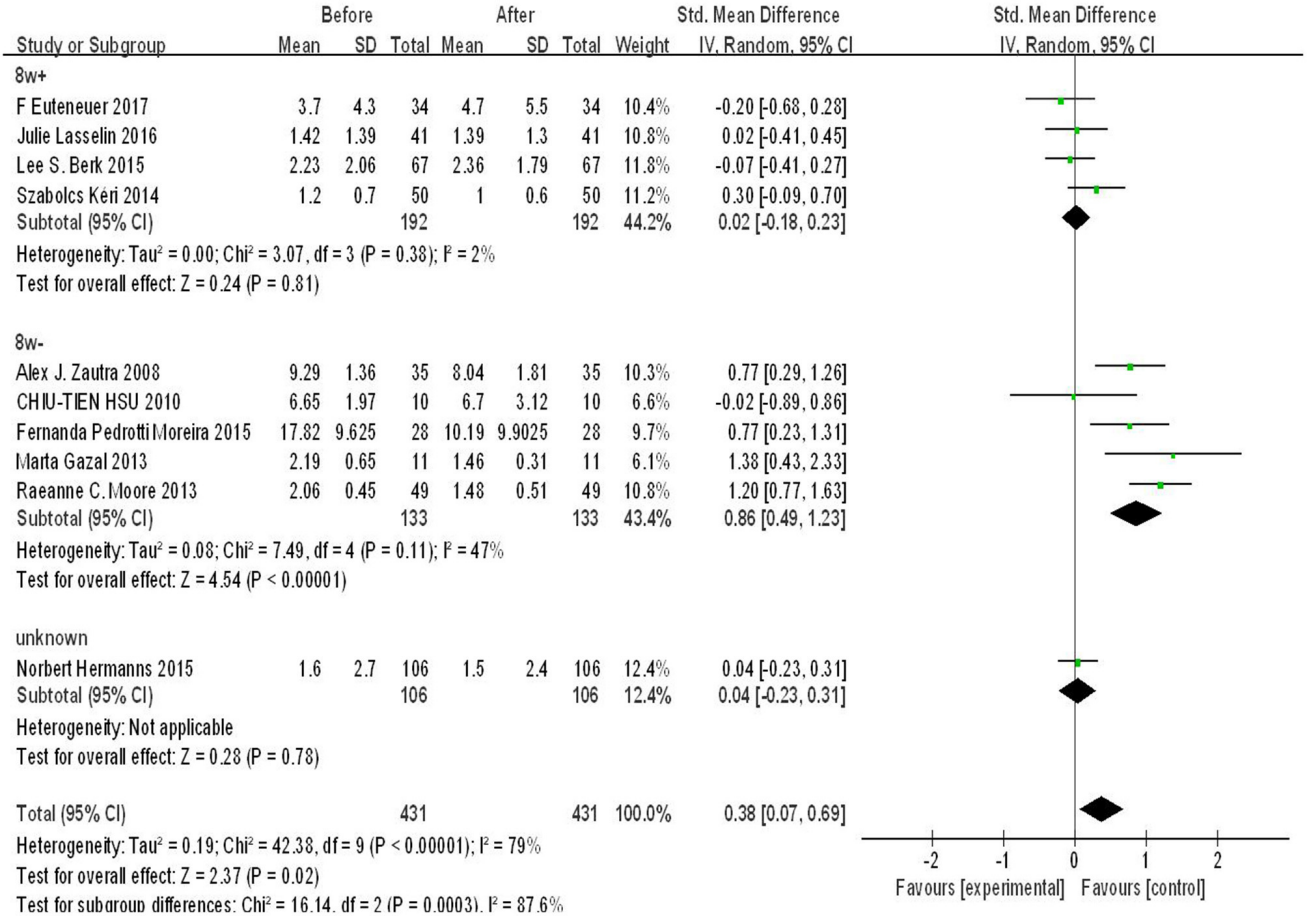
Forest plot for change in IL-6 before and after CBT with CBT intervention duration of 8 weeks.

According to the treatment duration of the studies included in our analysis, we used 8 weeks as a cut-off point to conduct subgroup analysis. Four original studies reported a treatment duration ≤ 8 weeks, five studies reported a treatment duration of more than 8 weeks, and one study did not report the treatment duration. As shown in Figure 3, there was significant difference in the subgroup ≤ 8 weeks (SMD = 0.86, 95%CI: 0.49, 1.23, *p* < 0.00001), whereas there was no significant difference in subgroup >8 weeks (*p* > 0.05). Moreover, the heterogeneity was lower in both of the two subgroups. There was strong heterogeneity and significant difference among subgroups (*p* = 0.0003, *I*^2^ = 87.6%).

**Figure 4:**
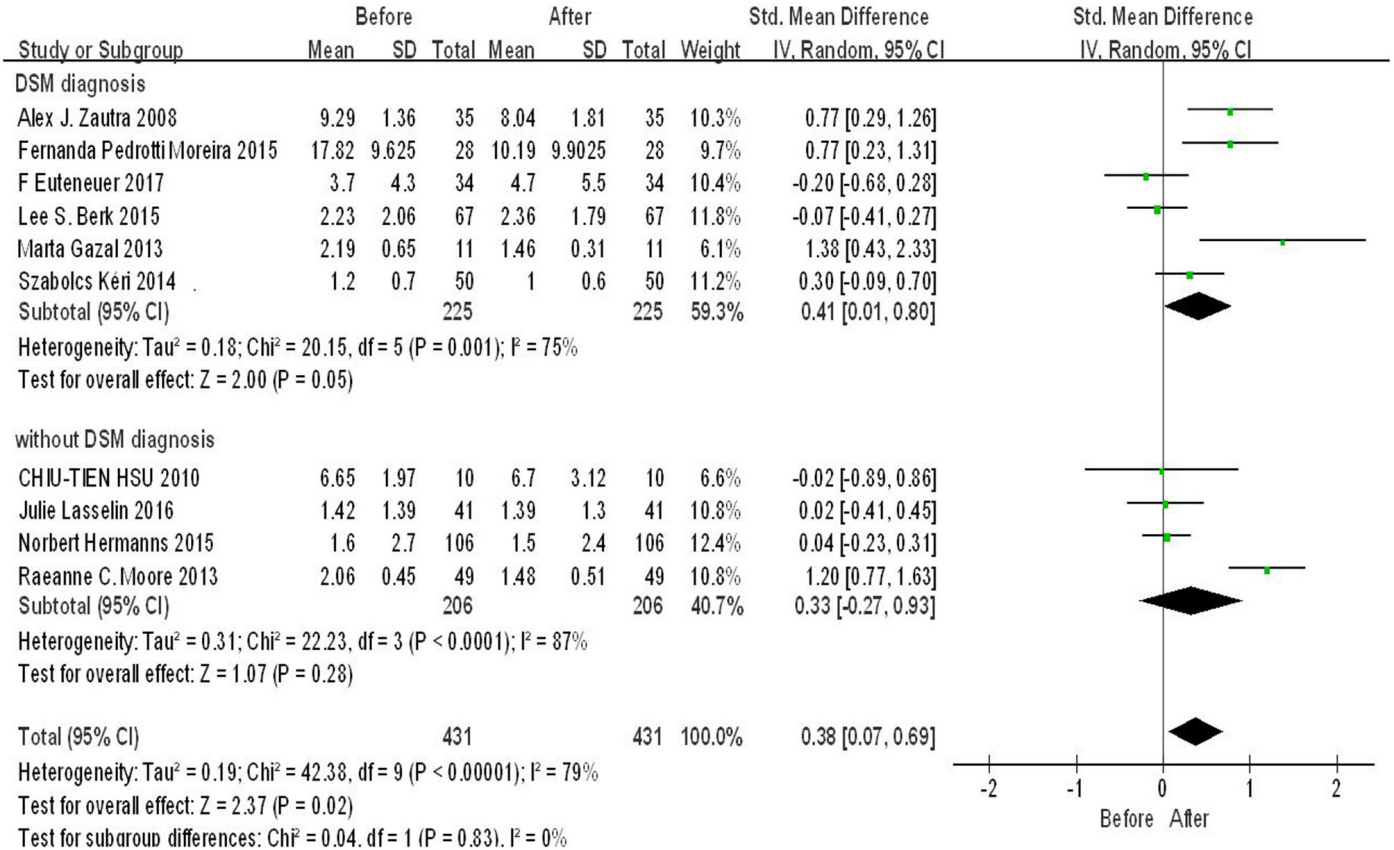
Forest plot for change in IL-6 before and after CBT under different depression diagnosis method.

According to the diagnosis method, we used diagnosis of depression as a cut-off point to conduct subgroup analysis. Six original studies reported depression diagnosed method is DSM, four reported others. As shown in Figure 4, there was significant difference in the subgroup DSM diagnosis (SMD = 0.41, 95%CI: 0.01, 0.80, *p* = 0.05), whereas there was no significant difference in subgroup without DSM diagnosis (*p* > 0.05). The heterogeneity was high in both of the two subgroups. There was low heterogeneity and significant difference among subgroups (*p* = 0.83, *I*^2^ = 0%).

### Meta-Regression Analysis

We conducted univariate meta-regression analysis for the year of publication, average age and sex ratio (male/female) of the included studies (Supplementary Table 1). Among them, the analysis results of the year of paper publication were statistically significant [β = −0.49, 95% CI = (−0.88, −0.09), *t* = −2.86, *p* = 0.02], and the goodness-of-fit of the model was good (Tau^2^ = 1.423). The estimation of variation among studies was relatively high (Adjusted R-squared = 52.91%).

### Sensitivity Analysis and Publication Bias

In order to test the stability of the results, we conducted sensitivity analysis to test the stability of the combined effect sizes, and their 95% CI. As shown in Supplementary Figure 4, the point estimate after the deletion of Moore et al. (34) fell outside the 95% CI of the total effect size. After the deletion, the estimate value was 0.26, and 95% CI = (−0.003, 0.532); therefore, the study may have impact on the pooled effect size. The Egger's test and Begg's test were used to evaluate the possibility of publication bias. The results indicated there was no potential publication bias for all included studies according to Egger's 95% CI = (−1.91, 7.48), *t* = 1.37, *p* = 0.208. In addition, the results of Begg's test did not show publication bias with *z* = 1.34, *p* = 0.180.

## Discussion

The results of our analysis indicate that CBT is associated with significant decreases of peripheral IL-6 levels with small effect in persons with depressive symptoms or MDD. Due to the potential heterogeneity of main outcome, subgroup analysis and meta-regression analysis were used to identify probable sources of heterogeneity. Subgroup analysis revealed a significant decrease in peripheral IL-6 in studies of 8 weeks duration or less, with no effect noted in studies of >8 weeks duration. The association between CBT and change in peripheral IL-6 was delimited to those studies that codified the diagnosis of MDD using the DSM (i.e., DSM-IV, DSM-IV-TR, or DSM-V). It was also revealed that publication year might be a potential contributor to heterogeneity in the findings. Moreover, no potential publication bias was identified in the studies in our analysis.

In general, our meta-analysis identified the potential modulating effect of CBT on IL-6. Cognitive behavioral therapy covers a range of strategies that could account for its potential anti-inflammatory effects. Cognitive behavioral therapy can trigger positive lifestyle changes that in turn reduce inflammation (36). Cognitive behavioral therapy also encourages the teaching of different relaxation techniques as well as participation in enjoyable activities (24). According to (37), research shows that in a dose-dependent manner, consistent relaxation practice may have favorable benefits on numerous immunological responses. An objective of CBT is to alter information processing. It has been reported that individuals who experience more frequent positive events show lower log IL-6 stimulation production, and that small positive events in daily life may result in reduced inflammatory responses to immune challenges (38). This study also reported that the effects were stronger for those in the lowest quartile of positive event frequency, implying that a lack of optimism in daily life may have a significant impact on inflammation. Furthermore, interpersonal happy events were more likely than non-interpersonal positive events to predict lower IL-6 overall and lower fibrinogen in women (38).

Our results only identified the changes of peripheral IL-6 in individuals with DSM diagnosed MDD, no statistical changes were reported in individuals with depressive symptoms assessed by other scales. Major depressive disorder is heterogenous in phenomenology, illness trajectory, and pathoetiology (39). According to (40), some individuals with depression may be more likely to exhibit an inflammatory biotype. For instance, it has been reported that individuals with depression who have melancholic traits have a distinct inflammatory profile compared to individuals without melancholic features (41). Moreover, distinct inflammatory profiles may also be linked to different depression subtypes (42–44). Whether it is diagnosed as MDD, Depression subtypes, and the effect of disease progression on inflammatory response deserve further attention. Therefore, whether the diagnosis of MDD, depression subtypes or the disease progression affects the inflammatory response after CBT deserves further attention.

According to Lanquillon (45), there were significant decreases in C-reactive protein in both responders and non-responders (i.e., with or without a 50% reduction in depression measurement, respectively) receiving either pharmacologic or psychosocial interventions. However, it has been separately reported that peripheral IL-6 is reduced in intervention responders but not for intervention non-responders; a finding also replicated by Yoshimura et al. (46). The latter study revealed that IL-6 could act as a proxy to treatment response, and may account for heterogeneity or response within the sample.

Our subgroup analysis showed that only the studies with intervention of less than 8w had a significant decrease in peripheral IL-6, but the changes of peripheral IL-6 in studies with more than 8w had no statistical difference. It has been proposed that IL-6 hyperproduction may play a pathogenetic role in the immunological pathophysiology of major depression due to its critical involvement in the early phase of the immune response cascade. Increased IL-6 activity in severe depression may be linked to hypotransferrinemia, hyperhaptoglobinemia, and hyperactivity of the HPA axis, according to the findings (47). Thus, our results may present that the short-term CBT may have obvious effects on inflammatory response due to rapid improvement of depressive symptoms. However, we did not find the direct evidence to support why the long-term effects of CBT on peripheral IL-6 were not significant. It is worthy to explore the underlying mechanisms. The future researches should increase the time and frequency of follow-up to furtherly determine our findings.

It is also worth noting that we identified publication year as a source of heterogeneity in our analysis. It is a testable hypothesis that changes in diagnostic criteria and treatments over the past two decades accounts for this variability. It is also possible that refinement of the CBT model and its implementation over the past several decades may be contributing to the observed heterogeneity (48). Our current findings provide valuable evidence for exploring the role of IL-6 in individuals with MDD receiving CBT in the future researches.

Moreover, evidence indicated that women are 1.5 to two times more likely than males to develop depression, and the onset of depression increases during the childbearing years. This female preponderance has been observed to last into elderly life. Patients above the age of 75 had a lower prevalence of depression, which did not appear to be connected to their socioeconomic level. Comorbid diseases, serum IL-6, albumin, and age may all have a role in determining which patients are more likely to develop depression symptoms (49). Therefore, further validation of confounding factors is needed in future studies. In addition, CRP and its precursor, IL-6, are linked to an increased incidence of depression, according to research (49). Therefore, future studies need to analyze the role of CRP levels, taking into account its association with IL-6.

### Limitations

There are several limitations that affect interpretations and inferences of our study. Firstly, the sample size of this meta-analysis was relatively small (i.e., only 10 eligible studies were included in the data analysis). Secondly, studies included in our analysis had varying definitions of depression and five studies included individuals with subthreshold depression. Moreover, the sensitivity analysis indicated that our results had insufficient stability. Additionally, limited information in the included studies betrayed a thorough analysis of the sources of heterogeneity. As with all studies, we could not control for residual confounding effects. Finally, previous antidepressant treatment may also affect the levels of inflammatory factors such as IL-6, but we could not distinguish participants with prescribed medications or medication-free in the original studies.

## Conclusions

Our results indicate that individuals with MDD or depressive symptoms receiving CBT have lower peripheral IL-6 concentrations. It is unknown whether the change on peripheral IL-6 levels is simply an association or whether the relationship observed has predictive and/or moderational effects. We did not evaluate CBT-IL-6 association from the point of view of dimension-based outcomes. Future studies will need to determine whether elevated IL-6 levels identify a biotype more likely (or less likely) to respond to CBT treatment (50).

## Data Availability Statement

The original contributions presented in the study are included in the article/Supplementary Material, further inquiries can be directed to the corresponding author/s.

## Author Contributions

BC and FY conceived and designed the study. HM and JX performed the data extraction and statistical analysis. RM, BC, FY, and KT contributed to the discussion. HM, JX, and RL took the lead in writing the manuscript. All authors discussed the results and commented on the manuscript. All authors contributed to the article and approved the submitted version.

## Funding

This work was sponsored by the MOE (Ministry of Education in China) Project of Humanities and Social Sciences (Project No. 21YJCZH004) and Ph.D. Research Startup Fund of Southwest University (Project No. SWU019039). The funding agents had no role in the design and conduct of the study; collection, management, analysis, interpretation of the data; preparation, review, or approval of the manuscript. The authors alone are responsible for the content and writing of the paper.

## Conflict of Interest

RM has received research grant support from CIHR/GACD/Chinese National Natural Research Foundation; speaker/consultation fees from Lundbeck, Janssen, Purdue, Pfizer, Otsuka, Takeda, Neurocrine, Sunovion, Bausch Health, Novo Nordisk, Kris, Sanofi, Eisai, Intra-Cellular, NewBridge Pharmaceuticals, Abbvie. RM is a CEO of Braxia Scientific Corp. KT receives personal fees from Braxia Scientific Corp. The remaining authors declare that the research was conducted in the absence of any commercial or financial relationships that could be construed as a potential conflict of interest.

## Publisher's Note

All claims expressed in this article are solely those of the authors and do not necessarily represent those of their affiliated organizations, or those of the publisher, the editors and the reviewers. Any product that may be evaluated in this article, or claim that may be made by its manufacturer, is not guaranteed or endorsed by the publisher.
