# Supplementary material for: The Impact of Cognitive Behavioral Therapy on Peripheral Interleukin-6 Levels in Depression: A Systematic Review and Meta-Analysis
Source: Front Psychiatry. 2022 May 13;13:844176. doi: 10.3389/fpsyt.2022.844176 (PMC9136073; doi:10.3389/fpsyt.2022.844176)
Supplement: Supplementary file 1 [file Data_Sheet_1.pdf]

**Supplemental Table 1. Univariable meta-regression analysis**

| Variables | *β* | 95% CI | *t* | *p* |
|---|---|---|---|---|
| **Published year** | -0.49 | -0.88, -0.09 | -2.86 | 0.02 |
| **Mean age of subjects (year)** | 0.00 | -0.08, 0.09 | 0.09 | 0.93 |
| **Sex Ratio(male/female)** | -1.88 | -6.94, 3.18 | -0.88 | 0.41 |

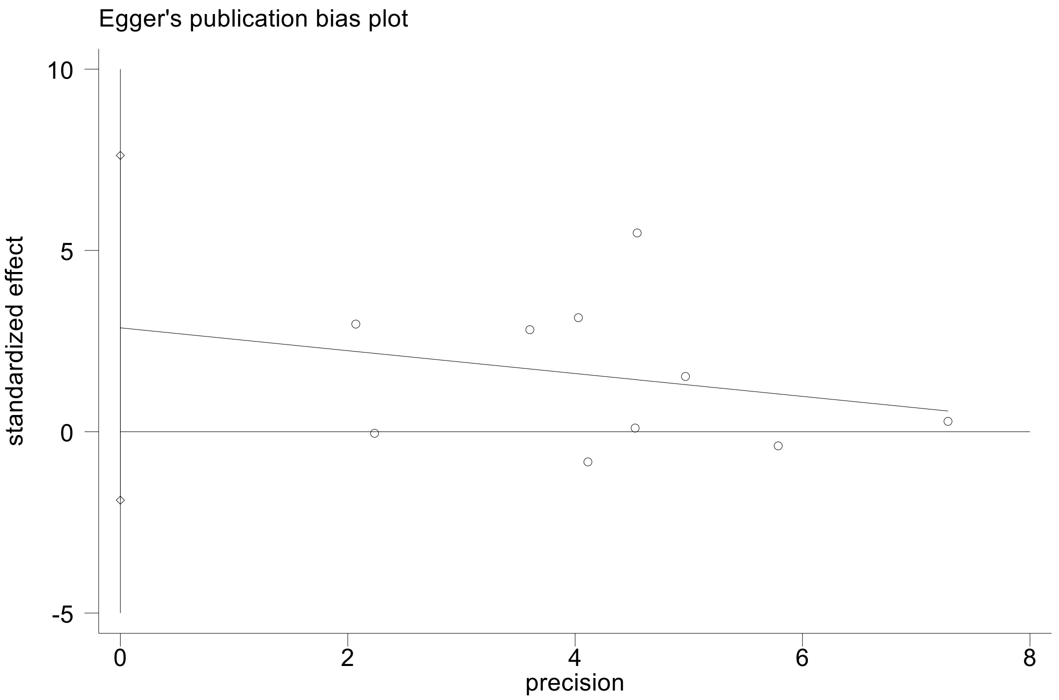


**Supplemental Figure 1. Egger' s publication bias plot**

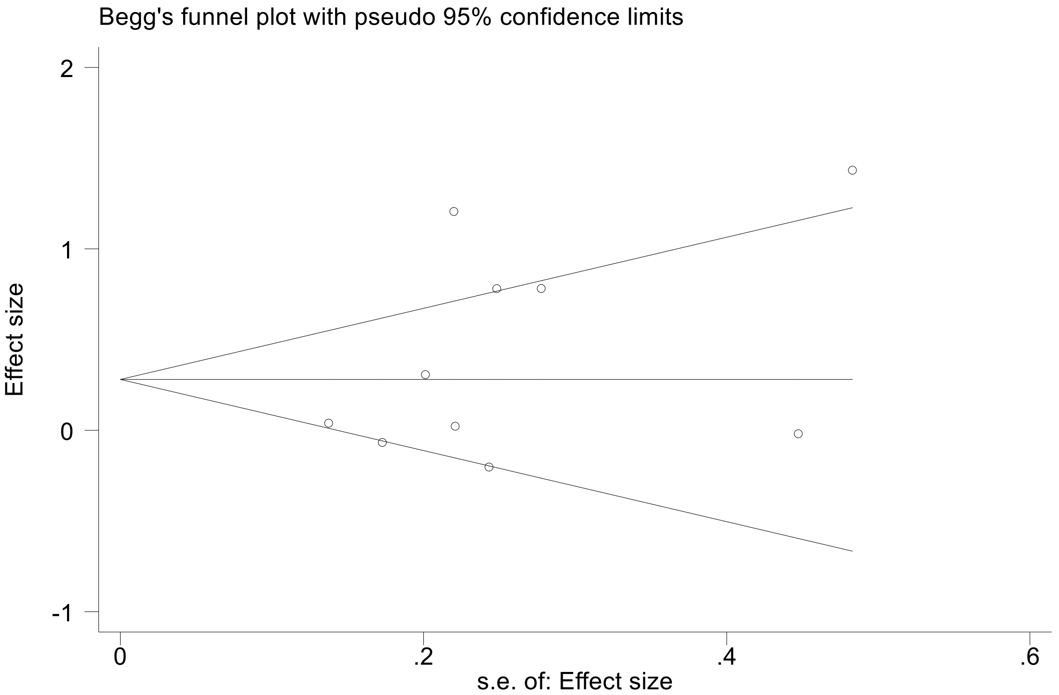


**Supplemental Figure 2. Begg' s funnel plot with pseudo 95% confidence limits**

| Study or Subgroup | Before Mean | Before SD | Before Total | After Mean | After SD | After Total | Weight | Std. Mean Difference IV, Random, 95% CI |
|---|---|---|---|---|---|---|---|---|
| **1.1.6 Underdeveloped** | | | | | | | | |
| CHIU-TIEN HSU 2010 | 6.65 | 1.97 | 10 | 6.7 | 3.12 | 10 | 6.6% | -0.02 [-0.89, 0.86] |
| Fernanda Pedrotti Moreira 2015 | 17.82 | 9.625 | 28 | 10.19 | 9.9025 | 28 | 9.7% | 0.77 [0.23, 1.31] |
| Marta Gazal 2013 | 2.19 | 0.65 | 11 | 1.46 | 0.31 | 11 | 6.1% | 1.38 [0.43, 2.33] |
| **Subtotal (95% CI)** | | | **49** | | | **49** | **22.3%** | **0.70 [0.02, 1.38]** |
| Heterogeneity: Tau² = 0.21; Chi² = 4.61, df = 2 (P = 0.10); I² = 57% | | | | | | | | |
| Test for overall effect: Z = 2.02 (P = 0.04) | | | | | | | | |
| **1.1.7 Developed** | | | | | | | | |
| Alex J. Zautra 2008 | 9.29 | 1.36 | 35 | 8.04 | 1.81 | 35 | 10.3% | 0.77 [0.29, 1.26] |
| F Euteneuer 2017 | 3.7 | 4.3 | 34 | 4.7 | 5.5 | 34 | 10.4% | -0.20 [-0.68, 0.28] |
| Julie Lasselin 2016 | 1.42 | 1.39 | 41 | 1.39 | 1.3 | 41 | 10.8% | 0.02 [-0.41, 0.45] |
| Lee S. Berk 2015 | 2.23 | 2.06 | 67 | 2.36 | 1.79 | 67 | 11.8% | -0.07 [-0.41, 0.27] |
| Norbert Hermanns 2015 | 1.6 | 2.7 | 106 | 1.5 | 2.4 | 106 | 12.4% | 0.04 [-0.23, 0.31] |
| Raeanne C. Moore 2013 | 2.06 | 0.45 | 49 | 1.48 | 0.51 | 49 | 10.8% | 1.20 [0.77, 1.63] |
| Szabolcs Kéri 2014 | 1.2 | 0.7 | 50 | 1 | 0.6 | 50 | 11.2% | 0.30 [-0.09, 0.70] |
| **Subtotal (95% CI)** | | | **382** | | | **382** | **77.7%** | **0.28 [-0.06, 0.63]** |
| Heterogeneity: Tau² = 0.18; Chi² = 33.09, df = 6 (P < 0.0001); I² = 82% | | | | | | | | |
| Test for overall effect: Z = 1.61 (P = 0.11) | | | | | | | | |
| **Total (95% CI)** | | | **431** | | | **431** | **100.0%** | **0.38 [0.07, 0.69]** |
| Heterogeneity: Tau² = 0.19; Chi² = 42.38, df = 9 (P < 0.00001); I² = 79% | | | | | | | | |
| Test for overall effect: Z = 2.37 (P = 0.02) | | | | | | | | |
| Test for subgroup differences: Chi² = 1.15, df = 1 (P = 0.28), I² = 12.9% | | | | | | | | |

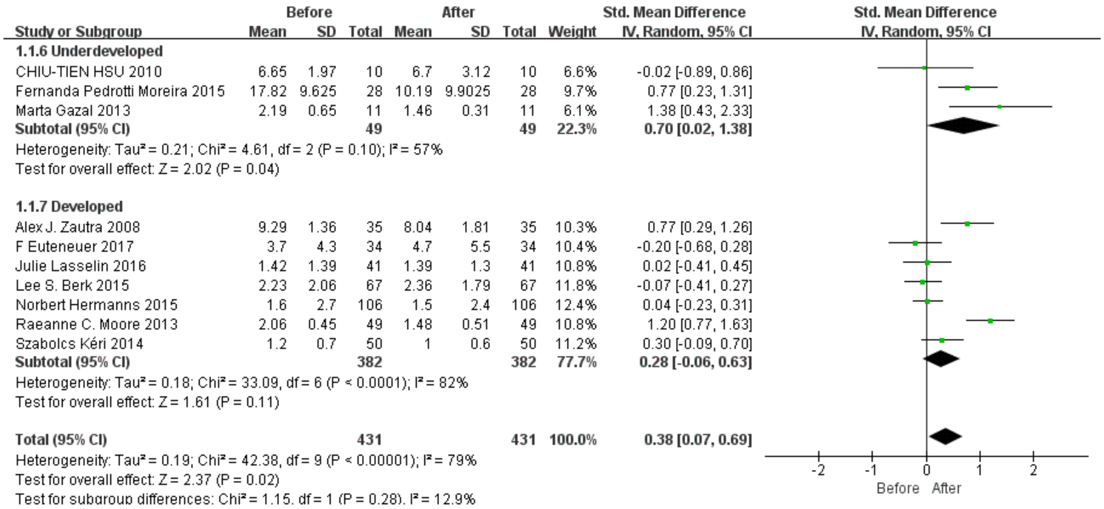


**Supplemental Figure 3. Forest plot for change in IL-6 before and after CBT under developed and underdeveloped countries**

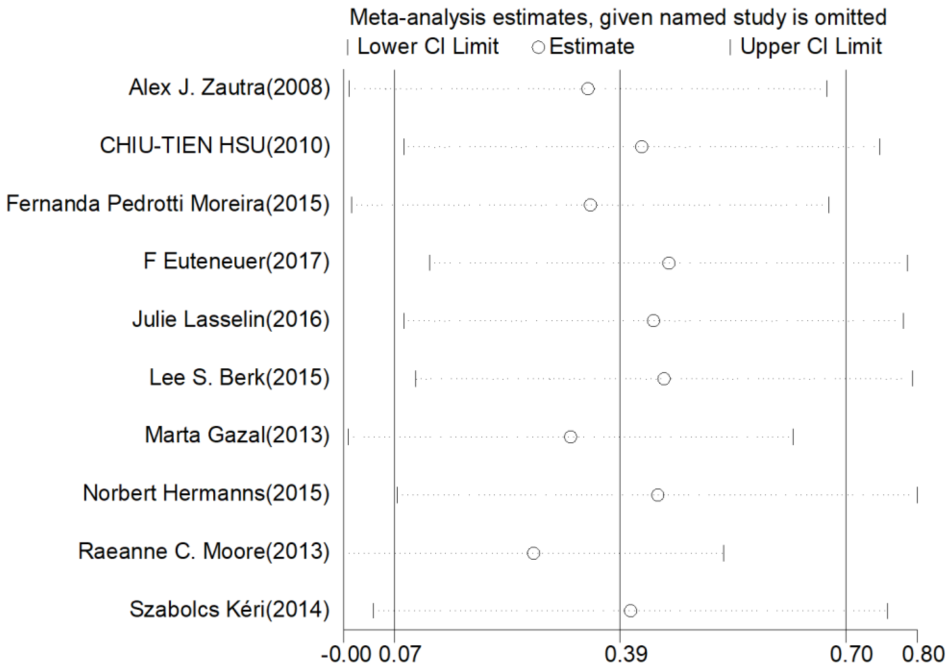


**Supplemental Figure 4. Results of sensitivity analysis**
